# Relationship between negative symptoms and neurocognitive functions in adolescent and adult patients with first-episode schizophrenia

**DOI:** 10.1186/s12888-016-1052-x

**Published:** 2016-10-06

**Authors:** Manli Huang, Yi Huang, Liang Yu, Jianbo Hu, Jinkai Chen, Pingbo Jin, Weijuan Xu, Ning Wei, Shaohua Hu, Hongli Qi, Yi Xu

**Affiliations:** 1 Department of Psychiatry, The First Affiliated Hospital, College of Medicine, Zhejiang University, The Key Laboratory of Mental Disorder’s Management of Zhejiang Province, No. 79, Qingchun Road, Hangzhou, 310003 China; 2 Department of General Surgery, The Second Affiliated Hospital, College of Medicine, Zhejiang University, No. 88, Jiefang Road, Hangzhou, 310000 China; 3 Department of Anesthesiology and Pain, Hang Zhou First People’s Hospital, No. 261, Huansha Road, Hangzhou, 310006 China

**Keywords:** Adolescent patients with first-episode schizophrenia, Correlation, Negative symptoms, Neurocognitive functions, PANSS

## Abstract

**Background:**

This study aimed to explore differences in links between negative symptoms and neurocognitive deficits in adolescent and adult patients with first-episode schizophrenia. Schizophrenia is a mental disorder often characterized by positive and negative symptoms, reduced emotional expression, excitatory status, and poor cognitive ability. The severity of negative symptoms in patients with schizophrenia was reported to be more related to poor quality of life, weak functional ability, and heavy burden from families than with the severity of positive symptoms. Previous studies suggested correlations between the severity of negative symptoms in patients with schizophrenia and neurocognitive deficits.

**Methods:**

This study included 92 patients (33 adolescents and 59 adults) with first-episode schizophrenia and 57 healthy people matched by age and education level. Neurocognitive functions and clinical symptoms were assessed using a standardized questionnaire.

**Results:**

Patients with first-episode schizophrenia showed neurocognitive deficits in most neuropsychological assessments compared with healthy people. With the variable of education level controlled, the negative factor score of adolescent patients with first-episode schizophrenia was strongly correlated with more time spent in part 1 (*r* = .646) and part 2 (*r* = .663) of the trail making test, and moderately correlated to more perseverative errors (*r* = .425) of the Wisconsin card sorting test and fewer correct trials 2 (*r* = −.425) of the continuous performance test. However, no such correlations were found in adult patients.

**Conclusions:**

This study indicated significant correlations between negative symptoms and most neurocognitive functions in patients with first-episode schizophrenia, with a stronger correlation in adolescent patients.

**Trial registration:**

The trial registration number is ChiCTR-COC-14005302, while retrospectively registered on January 5, 2014.

## Background

Schizophrenia is a mental disorder often described in terms of hallucination and abnormal social behavior, including poor rapport and social isolation. It affects about 0.3–0.7 % [1] of people at some point (the peak age of onset is 18–25 years [2]) in their lives and accounts for approximately 1 % of disability-adjusted life year all over the world [3]. Patients with schizophrenia have been reported to have abnormities in brain structure and function [1, 4]. The causes of schizophrenia have been widely investigated, and many studies have suggested genetics, prenatal and adolescent neurodevelopment, psychological health, and social living environment as common risk factors [2, 5, 6].

Schizophrenia is often characterized by positive and negative symptoms, reduced emotional expression, excitatory status, and poor cognitive ability. The positive and negative syndrome scale (PANSS) is a 30-item rating scale designed by Kay to assess the severity of schizophrenia symptoms. Literature research suggested that a five-factor model, which consists of positive, negative, disorganized–concrete, excited, and anxious–depressive factor, better captured the PANSS structure in patients with schizophrenia [7–14].

Generally, negative symptoms refer to poor emotional reactions or thought processes, including emotion impoverishment, speech barrier, thought inflexibility, and decreased activity. It has been reported that severity of negative symptoms in patients with schizophrenia was more related to poor quality of life [15], weak functional ability [16] and heavy burden from families, compared with the severity of positive symptoms [17]. Moreover, patients with schizophrenia having obvious negative symptoms often had a history of poor social adaptability before the onset of illness, and their responses to medication were often limited [18]. The present study focused on negative symptoms owing to their serious influence on human life.

Adolescence is a key period of neurological and psychological development. If psychosis occurs in one’s late adolescence when the frontal lobe is still developing, some extraordinary differences may exist in both neurocognitive deficits and clinical symptoms [19], compared with an adult patient with schizophrenia. A 5-year follow-up clinical study on very early-onset schizophrenia, using magnetic resonance imaging (MRI), showed damage processes in brain regions of adolescent patients with schizophrenia. However, adult cognitive deficits are known to be mediated by environmental factors [20]. A latest study also showed that adolescent patients with schizophrenia performed worse than adult patients in tasks of working memory, language, and motor function [21].

In addition, previous studies have suggested correlations between negative symptoms in patients with schizophrenia and neurocognitive deficits involving intelligence, executive function, sustained-attention function, sensory motor function [22], cognitive function [23], and memory [22, 24]. Patients with higher negative scores of PANSS had more perseverative errors (PE) and fewer completed categories (CC) in the Wisconsin card sorting test (WCST) (standing for executive function) [25], and poorer performance in University of Pennsylvania Smell Identification Test (standing for function of an individual’s olfactory system) [26]. They also experienced more difficulties in the trail making test (TMT) (standing for visual attention and executive function), verbal fluency test (standing for semantic memory) [25], and faux pas test (standing for sociability) [27].

As mentioned, correlations exist between negative symptoms in patients with schizophrenia and neurocognitive deficits. To get rid of clinical interference, we aimed to patients with first-episode schizophrenia who were drug naive. Thomas R. Insel pointed out that schizophrenia should be regarded a collection of neurodevelopmental disorders [2]. Since both symptoms and neurocognitive deficits may be caused by neurodevelopmental problems in adolescent patients with first-episode schizophrenia whose prefrontal cortices are under development [28–30], it is assumed that their negative symptoms are more closely related to neurocognitive deficits than those of adult patients with first-episode schizophrenia having developed prefrontal cortices. If the aforementioned hypothesis is correct, the negative symptom scores of adolescent patients with schizophrenia become more important in evaluating neurocognitive deficits, compared with adult patients with first-episode schizophrenia. It is easy for psychiatrists to obtain patients’ PANSS scores, while evaluation of neurocognitive functions is complicated and time-consuming. That is to say, lower negative symptom scores indicate stronger neurocognitive deficits in adolescent patients with schizophrenia. In addition, improvement of patients’ negative symptoms can be used in evaluating the therapeutic effect.

The present study had two objectives (the first objective was replication of the objective of previous studies to some degree, while the second objective was novel):

1. To evaluate neurocognitive deficits and clinical symptoms of patients with first-episode schizophrenia.
2. To study the relationship between negative symptoms and neurocognitive functions in patients with first-episode schizophrenia, especially adolescent patients.

## Methods

### Study subjects

This study included 92 patients with first-episode schizophrenia and 57 healthy volunteers. All the patients were divided into two groups—33 adolescent patients with first-episode schizophrenia (whose age and age of onset were less than 18 years) and 59 adult patients with first-episode schizophrenia (whose age and age of onset were equal to or more than 18 years).

Patients were recruited from the First Affiliated Hospital of Medicine School of Zhejiang University, who fulfilled diagnostic criteria for schizophrenia in the *Diagnostic and Statistical Manual of Mental Disorders*, Fourth Edition (DSM-IV). The Structured Clinical Interview for DSM-IV-TR, routine laboratory tests, and physical and neurological examinations were all administered to each participant.

Inclusion criteria for patient selection were as follows: (1) age between 13 and 45 years; (2) having a DSM-IV diagnosis of schizophrenia; (3) experiencing their first episode; (4) being drug naive; (5) both males and females; and (6) ethnicity of Han origin. (Since the establishment of People’s Republic of China, the national government officially identifies 56 distinct ethnic groups according to different dresses, music types, and languages, the largest of which is the Han Chinese. Everybody in China belongs to only one ethnic group that is marked on his/her identity card. Different ethnic groups mean different cultures and cognitive abilities. The patients in the present study all belonged to Han Chinese within three generations. In this way, the interference of ethnic groups could be reduced.) Patients were excluded if they fell into any of the following categories: (1) having a history or presence of any severe unstable systemic disease; (2) having a history of organic brain disease, cerebral trauma, seizure disorder, mental retardation, or MRI evidence of structural brain abnormalities; and (3) being pregnant, lactating, or planning to be pregnant within 6 months.

Volunteers through advertising served as normal controls. None of the controls had a family history of mental disorder or a medical history of somatic or organic brain disease, cerebral trauma, severe mental retardation, or MRI evidence of structural brain abnormalities. All volunteers accepted MRI performed by experienced professional staff in the First Affiliated Hospital of Medical School of Zhejiang University using the Philips Magnetic Resonance Imaging Systems Achieva 3.0T TX (Philips Healthcare, Netherlands). The system was tested for data stability before use. MRI was completed within 1 week after neuropsychological tests. The age and education level of the controls were matched to those of the patients. The controls were of the same ethnic Han origin.

The study was approved by the ethics committee of the First Affiliated Hospital of Medical School of Zhejiang University, and conducted in accordance with the Code of Ethics of the World Medical Association (Declaration of Helsinki). All subjects and their legal guardians (applicable if participants were less than 16 years old) provided written informed consent before participating in the study.

The tests were completed for all subjects by fully trained psychiatrists with consistent training courses in the First Affiliated Hospital of Medical School of Zhejiang University. The inter-rater agreement of PANSS, WCST, continuous performance test (CPT), TMT and Stroop color-word test (SCWT) was measured, and the kappa coefficient was 0.80, 0.82, 0.78, 0.83, and 0.84, respectively. To reduce errors, all items were assessed in the morning and neuropsychological tests were administered first. To minimize the potential of order effects, the sequence of the neuropsychological tests was randomized. The tests usually lasted 1–2 h and were completed within 1 day for healthy people and two consecutive days for patients. Clinical assessments were rated on the same days as the aforementioned neuropsychological tests.

### Neuropsychological assessments

Four neuropsychological assessments including WCST, CPT, TMT, and SCWT were used in all 149 subjects (92 patients with first-episode schizophrenia and 57 healthy controls). All of them were typical tests for patients with schizophrenia in evaluating neurocognitive deficits standing for executive function; sustained, visual, and selective attention; and so forth. In this study, standard Chinese versions of neuropsychological assessments were used, and the rater was blinded to grouping.

#### Wisconsin card sorting test (modified version)

The WCST was a cognitive task that predominantly assessed executive function because of its reported sensitivity to frontal lobe dysfunction [31]. Subjects were asked to sort 48 cards on the basis of three possible categories (color, number, and shape). After six consecutive correct responses, the subjects were asked to change the sorting principle to another category. The test ended when the subjects completed all six categories correctly or used 48 cards. The total trials (TT), correct trials (CT), total number of errors (TE), PE, random errors (RE), and number of CC were recorded [32].

#### Continuous performance test

The CPT measured the subjects’ sustained and selective attention. It was divided into three parts: (1) different numbers appeared on the screen one by one, and the mouse was clicked when “4” appeared; (2) eight numbers appeared on the screen at the same time, and the mouse was clicked when “4” appeared; and (3) eight numbers appeared on the screen at the same time, and the mouse was clicked when “7” appeared. Each number remained on the screen for 150 ms, and the interval time was 550 ms. The subjects were asked to finish all the three parts. The correct trials of three parts (CPT 1, CPT 2, and CPT 3) and the perseverative errors in part 3 (PE of CPT 3) were recorded [33].

#### Trail making test

The TMT was a neuropsychological test of visual attention and task switching, which provided information about visual search speed, processing speed, mental flexibility, and executive functioning. It consisted of two parts in which the subjects were instructed to connect a set of 25 dots as fast and accurately as possible. In part 1 of TMT, the subjects were required to quickly draw lines to connect consecutively numbered circles. In part 2, the subjects were asked to alternately combine numbers with different colors in ascending order [34]. The task completion was measured in seconds and recorded as TMT 1 and TMT 2, respectively, for parts 1 and 2. TMT 1 was used to examine cognitive processing speed, while TMT 2 referred to executive functioning [35].

#### Stroop color-word test

The SCWT, also called Stroop test, measured selective attention, cognitive flexibility, and processing speed. It was used as a tool in evaluating executive functions. In step 1 of SCWT (SCWT 1), the subjects were asked to read out three black words as fast as possible, which stood for a certain color. Then, in step 2 (SCWT 2), they were instructed to tell the color of three color parcels as fast as possible. Finally, in step 3 (SCWT 3), they were required to tell the color of three color words as fast as possible, while each color was different from the word’s meaning. The performance for each condition was calculated from the processing time per item in seconds. The reaction time (RT of SCWT) difference in part 3 relative to part 2 was called the “interference” effect [36].

### Clinical assessments

The PANSS [37] was used to assess the clinical symptom severity in 92 patients with first-episode schizophrenia in the time of the neuropsychological tests. The PANSS was a 30-item medical scale, originally grouped into 3 parts, including 7 positive symptoms (i.e., delusions, conceptual disorganization, hallucinations, hyperactivity, grandiosity, suspiciousness/persecution, and hostility), 7 negative symptoms (i.e., blunted affect, emotional withdrawal, poor rapport, passive/apathetic social withdrawal, difficulty in abstract thinking, lack of spontaneity and flow of conversation, and stereotyped thinking) and 16 general psychopathology items (i.e., somatic concern, anxiety, guilt feelings, tension, mannerisms and posturing, depression, motor retardation, uncooperativeness, unusual thought content, disorientation, poor attention, lack of judgment and insight, disturbance of volition, poor impulse control, preoccupation, and active social avoidance) that assessed the degree of psychopathology on a number of symptomatic domains. Each item was rated from 1 (no evidence) to 7 (extreme) based on objective criteria [37]. In this study, standard Chinese versions of PANSS were used to evaluate symptoms of 92 patients with first-episode schizophrenia.

In recent years, the five-factor model has been widely used in different schizophrenia research areas, including cognitive function [14] and insight [38]. With the outcome of principal component analysis and reliability analysis, SI Tianmei suggested that the structure, validity, and reliability of PANSS (Chinese version) were acceptable, and put forward a five-factor model of Chinese-version PANSS, including negative, positive, excitement–hostility, anxiety depression, and cognitive defect factors [39]. In the study, the aforementioned five-factor model was used.

### Statistical analyses

All data were expressed as means ± standard deviation. An unpaired *t* test and a chi-square test were used to compare the general conditions and neuropsychological assessment outcomes between patients and healthy people. The relationship between general conditions and neuropsychological assessments of patients was computed using a correlational analysis. A partial correlation was determined to control the potential influence of education level on the relationship between the negative factor scores and neuropsychological assessments. The difference in correlation coefficients between adolescent and adult patients with first-episode schizophrenia was computed by a Fisher’s *Z* test. The false discovery rate was used to control the inflated Type I error rate. To get convincing results, all statistical tests were two-tailed and a *p*-value < .05 was considered to be significant.

All analyses were carried out using the Statistical Package for the Social Sciences (SPSS) version 18.0 for Windows (IBM, IL, USA).

## Results

### Demographics, neuropsychological assessments, and clinical assessments

No significant difference was found in age (*t* = −.697, *p* = .487), education level (*t* = −1.832, *p* = .070), or gender (*χ* ^2^ = .129, *p* = .625) between patients with first-episode schizophrenia and healthy people.

In the WCST, the mean scores of CT and CC for patients with schizophrenia were lower than those of controls, while the mean scores of TE, PE, and RE for patients with schizophrenia were higher than those of controls (all *p-*values < .01). However, no significant difference was observed in TT (*t* = .763, *p* = .447).

The results of TMT and SCWT were similar to those of WCST. Compared with the controls, patients with schizophrenia spent more time in TMT 1 (*t* = 2.460, *p* = .015), TMT 2 (*t* = 2.777, *p* = .006), SCWT 2 (*t* = 6.574, *p* < .001), SCWT 3 (*t* = 7.867, *p* < .001) and RT of SCWT (*t* = 5.000, *p* < .001). In the CPT, patients with first-episode schizophrenia got a higher mean score of PE of CPT 3 compared with healthy people (*t* = 2.022, *p* = .045). The details are displayed in Table 1.

**Table 1:** Demographic, neuropsychological assessments and clinical assessments outcomes of first-episode schizophrenics and healthy people

		First-episode schizophrenics (*N* = 92)	Healthy people (*N* = 57)	t/*χ* ^2^	*P*-value
Age (years)		22.86 ± 7.82	23.84 ± 8.69	-.697	.487
Education level (years)		10.77 ± 3.01	11.77 ± 3.38	-1.832	.070
Duration of illness (months)		12.26 ± 18.37	None	None	None
Gender (male/total)		36/92	24/57	.129	.625
PANSS	T	84.18 ± 16.71	None	None	None
	P	23.88 ± 7.37	None	None	None
	N	19.30 ± 8.12	None	None	None
	G	41.00 ± 8.32	None	None	None
WCST	TT	47.34 ± 2.73	47.04 ± 2.08	.763	.447
	CT	25.42 ± 10.47	33.91 ± 7.30	-5.821	< .001
	TE	21.80 ± 10.56	12.77 ± 8.18	5.846	< .001
	PE	13.82 ± 7.54	7.56 ± 4.99	6.089	< .001
	RE	7.96 ± 5.00	5.49 ± 3.94	3.342	.001
	CC	2.87 ± 2.06	4.68 ± 1.68	-5.872	< .001
CPT	CPT 1	11.01 ± 2.27	11.18 ± .80	-.635	.527
	CPT 2	11.63 ± 3.33	12.12 ± 1.65	-1.201	.232
	CPT 3	11.95 ± 2.99	12.23 ± 1.28	-.795	.428
	PE of CPT 3	.84 ± 1.75	.37 ± 1.08	2.022	.045
TMT	TMT 1	58.13 ± 34.06	43.56 ± 35.77	2.460	.015
	TMT 2	114.31 ± 62.47	84.51 ± 64.39	2.777	.006
SCWT	SCWT 1	50.82 ± 15.19	44.96 ± 21.87	1.772	.080
	SCWT 2	90.16 ± 33.32	62.51 ± 17.90	6.574	< .001
	SCWT 3	143.27 ± 49.19	97.51 ± 22.82	7.687	< .001
	RT of SCWT	53.10 ± 29.27	35.00 ± 14.72	5.000	< .001

Values are the means ± SD. All *P*-values were compared with Healthy people analyzed by an unpaired *t*-test

*T* PANSS total scores, *P* positive syndrome scale scores, *N* negative syndrome scale scores, *G* general psychopathology syndrome scale scores, *TT* total trials, *CT* correct trials, *TE* total number of errors, *PE* perseverative errors, *RE* random errors, *CC* the number of completed categories, *PE of CPT 3* perseverative errors of CPT 3, *RT of SCWT* reaction time of SCWT;

The mean score of CPT 2 for adolescent patients with first-episode schizophrenia was lower than that of adults (*t* = −2.064, *p* = .043). However, no other significant difference was noted in clinical or neuropsychological assessments between adolescent and adult patients with first-episode schizophrenia. The details are displayed in Table 2.

**Table 2:** Demographic, neuropsychological assessments and clinical assessments outcomes of adolescent and adult first-episode schizophrenics

		Adolescent first-episode schizophrenics (*N* = 33)	Adult first-episode schizophrenics (*N* = 59)	t	*P*-value
Age (years)		15.48	25.02	−10.107	< .001
Education level (years)		9.61	11.42	−3.518	.001
Duration of illness (months)		9.67 ± 1.50	11.42 ± 3.43	−1.182	.240
PANSS	T	85.30 ± 18.04	83.56 ± 16.04	.462	.645
	P	23.33 ± 7.21	24.19 ± 7.50	-.537	.593
	N	20.45 ± 10.26	18.66 ± 6.65	.904	.371
	G	41.52 ± 8.70	40.71 ± 8.16	.434	.666
WCST	TT	47.64 ± 1.11	47.17 ± 3.30	.990	.325
	CT	24.06 ± 9.62	26.19 ± 10.92	-.968	.336
	TE	23.27 ± 10.15	20.98 ± 10.78	1.015	.314
	PE	14.64 ± 6.28	13.36 ± 8.18	.840	.403
	RE	8.55 ± 5.10	7.63 ± 4.95	.837	.406
	CC	2.64 ± 2.07	3.00 ± 2.05	-.810	.421
CPT	CPT 1	10.79 ± 2.30	11.14 ± 2.25	-.700	.486
	CPT 2	10.67 ± 3.45	12.17 ± 3.16	−2.064	.043
	CPT 3	11.61 ± 3.65	12.14 ± 2.57	-.738	.464
	PE of CPT 3	.61 ± 1.48	.97 ± 1.88	−1.013	.314
TMT	TMT 1	57.85 ± 30.69	58.28 ± 36.06	-.061	.951
	TMT 2	104.68 ± 48.052	119.69 ± 69.05	−1.223	.225
SCWT	SCWT 1	50.50 ± 16.44	50.99 ± 14.59	-.145	.885
	SCWT 2	88.25 ± 30.03	91.23 ± 35.23	-.430	.669
	SCWT 3	134.98 ± 33.04	147.90 ± 55.98	−1.391	.168
	RT of SCWT	46.74 ± 19.66	56.66 ± 33.09	−1.804	.075

Values are the means ± SD. All *P*-values were compared with Healthy people analyzed by an unpaired *t*-test

*T* PANSS total scores, *P* positive syndrome scale scores, *N* negative syndrome scale scores, *G* general psychopathology syndrome scale scores, *TT* total trials, *CT* correct trials, *TE* total number of errors, *PE* perseverative errors, *RE* random errors, *CC* the number of completed categories, *PE of CPT 3* perseverative errors of CPT 3, *RT of SCWT* reaction time of SCWT;

### Correlational analysis between clinical assessments and neuropsychological assessments in patients with first-episode schizophrenia

Using the five-factor model of PANSS, the negative factor scores were computed using an average of nine items (i.e., Blunted Affect, Emotional Withdrawal, Poor Rapport, Passive/Apathetic Social Withdrawal, Lack of Spontaneity and Flow of Conversation, Motor Retardation, Disturbance of Volition, Preoccupation, and Active Social Avoidance). Pearson correlational analysis was performed to assess the relationship between general conditions, negative symptoms, and neuropsychological assessments outcomes.

Considering the general conditions, significant correlations were found between the educational level and neuropsychological assessments on nine items, especially on TMT 1 (*r* = −.462, *p* < .001), TMT 2 (*r* = −.473, *p* < .001), SCWT 1 (*r* = −.406, *p* < .001), and SCWT 2 (*r* = −.432, *p* < .001). In addition, it has been reported in a previous study that sustained attention, ideation fluency, cognitive flexibility, and visuospatial memory had a positive correlation with education in deficit schizophrenia [40]. Partial correlation analysis was used to get rid of the interference. With the variable of education level controlled, persuasive results were obtained. In total, significant correlations were observed between the negative factor scores and neuropsychological assessments on 12 items (i.e., CT, TE, PE, RE and CC of WCST; CPT 1; TMT 1 and TMT 2; SCWT 1, SCWT 2, SCWT 3, and RT of SCWT). Moreover, the negative factor score was moderately correlated to SCWT 1 (*r* = .410, *p* < .001) and SCWT 3 (*r* = .409, *p* < .001). The details are displayed in Table 3.

**Table 3:** The correlation and partial correlation (negative factor) matrix of general conditions, negative symptoms and neuropsychological assessments outcomes on first-episode schizophrenics

		Age	Age of onset	Education level	Duration of illness	Negative factor
WCST	TT	-.032	-.030	.004	-.011	-.010
	CT	.044	-.028	.276**	-.075	-.381**
	TE	-.045	.028	-.271**	.078	.381**
	PE	-.034	.043	-.257*	.086	.272**
	RE	-.033	-.001	-.159	.002	.397**
	CC	-.028	-.084	.283**	-.044	-.290**
CPT	CPT 1	.242*	.179	.141	.251*	-.230*
	CPT 2	.187	.192	-.044	.148	-.178
	CPT 3	.040	.002	.056	-.090	-.080
	PE of CPT 3	.051	.099	-.007	.024	-.045
TMT	TMT 1	-.009	.175	-.462**	.016	.306**
	TMT 2	.043	.222*	-.473**	.014	.293**
SCWT	SCWT 1	.087	.156	-.406**	.055	.410**
	SCWT 2	-.067	.087	-.432**	.072	.331**
	SCWT 3	-.063	-.013	-.206*	-.104	.409**
	RT of SCWT	-.029	-.120	.145	-.256*	.335**

False Discovery Rate: *p* (CT) < *p* (TE) < *p* (RE) < *p* (SCWT 1) < *p* (SCWT 3) < *p* (SCWT 2) < *p* (RT of SCWT) < *p* (TMT 1) < *p* (CC) < *p* (TMT 2) < *p* (PE) < *p* (CPT 1) < .05 < *p* (CPT 2) < *p* (CPT 3) < *p* (PE of CPT 3) < *p* (TT), while *p* (CPT 1) = .028 < .05*12/16 = .0375

|r|∈[.8,1] means highly strong correlation, |r|∈[.6,.8) means strong correlation, |r|∈[.4,.6) means moderate correlation, |r|∈[.2,.4) means weak correlation, |r|∈[0,.2) means very weak correlation or no correlation

*TT* total trials, *CT* correct trials, *TE* total number of errors, *PE* perseverative errors, *RE* random errors, *CC* the number of completed categories; *PE of CPT 3* perseverative errors of CPT 3, *RT of SCWT* reaction time of SCWT

*P < .05, **P < .01, the significance of the correlation coefficient

### Comparison of adolescent and adult patients with first-episode schizophrenia

In both adolescent and adult patients with schizophrenia, the negative factor score was correlated with more time spent in part 1 (*r* = .527, *p* = .002; *r* = .342, *p* = .009), part 2 (*r* = .400, *p* = .023; *r* = .365, *p* = .005), and part 3 (*r* = .552, *p* = .001; *r* = .452, *p* < .001) of SCWT, a lower mean score of CT (*r* = −.486, *p* = .005; *r* = −.313, *p* = .017), higher mean scores of TE (*r* = .489, *p* = .005; *r* = .297, *p* = .024), and RE of WCST (*r* = .488, *p* = .005; *r* = .310, *p* = .018). No significant difference was found in the aforementioned items of correlation coefficients (all *p*-values > .05) between the two groups. The details are displayed in Table 4.

**Table 4:** The comparison of correlation coefficients between adolescent and adult first-episode schizophrenics

		Adolescent (*N* = 33)		Adult (*N* = 59)		u-value	*P*-value
		r	z	r	z		
WCST	CT	-.486**	-.531	-.313*	-.324	-.915	.358
	TE	.489**	.535	.297*	.306	1.010	.312
	RE	.488**	.533	.310*	.321	.941	.347
SCWT	SCWT 1	.527**	.586	.342**	.356	1.015	.308
	SCWT 2	.400*	.424	.365**	.383	.181	.857
	SCWT 3	.552**	.621	.452**	.487	.592	.555

*CT* correct trials, *TE* total number of errors, *RE* random errors

*P < .05, **P < .01, the significance of the correlation coefficient

In adolescent patients with schizophrenia, the negative factor score was strongly correlated with more time spent in TMT 1 (*r* = .646, *p* < .001) and TMT 2 (*r* = .663, *p* < .001), and moderately or weakly correlated with a higher mean score of PE (*r* = .425, *p* = .015) of WCST and lower mean scores of CPT 2 (*r* = −.425, *p* = .015). However, no correlation between the aforementioned items could be found in adult patients.

The negative factor score was weakly correlated with a lower mean score of CPT 3 (*r* = −.385, *p* = .030) in adolescent patients with schizophrenia, while it was weakly correlated with a higher mean score of CPT 3 (*r* = .303, *p* = .021) in adults. The details are displayed in Table 5.

**Table 5:** The correlation matrix of negative factor scores and the neuropsychological test measures on adolescent and adult first-episode schizophrenics

		Negative factor scores		
		Total	Adolescent	Adult
WCST	TT	-.010	.050	-.059
	CT	-.381**	-.486**	-.313*
	TE	.381**	.489**	.297*
	PE	.272**	.425*	.199
	RE	.397**	.488**	.310*
	CC	-.290**	-.352	-.214
CPT	CPT 1	-.230*	-.368	-.094
	CPT 2	-.178	-.425*	.081
	CPT 3	-.080	-.385*	.303*
	PE of CPT 3	-.045	.221	-.150
TMT	TMT 1	.306**	.646**	.101
	TMT 2	.293**	.663**	.173
SCWT	SCWT 1	.410**	.527**	.342**
	SCWT 2	.331**	.400*	.365**
	SCWT 3	.409**	.552**	.452**
	RT of SCWT	.335**	.318	.412**

Adolescent False Discovery Rate: *p* (TMT 1) < *p* (TMT 2) < *p* (SCWT 3) < *p* (SCWT 1) < *p* (CT) < *p* (TE) < *p* (RE) < *p* (PE) < *p* (CPT 2) < *p* (SCWT 2) < *p* (CPT 3) < *p* (CPT 1) < *p* (CC) < .05 < *p* (RT of SCWT) < *p* (PE of CPT 3) < *p* (TT), while *p* (CPT 3) = .03 < .05*11/16 = .034 and *p* (CPT 1) = .038 > .05*12/16 = .0375. So CC and CPT 1 are not significant

Adult False Discovery Rate: *p* (SCWT 3) < *p* (RT of SCWT) < *p* (SCWT 2) < *p* (SCWT 1) < *p* (CT) < *p* (RE) < *p* (CPT 3) < *p* (TE) < .05 < *p* (CC) < *p* (PE) < *p* (TMT 2) < *p* (PE of CPT 3) < *p* (TMT 1) < *p* (CPT 1) < *p* (CPT 2) < *p* (TT), while *p* (TE) = .024 < .05*8/16 = .025

|r|∈[.8,1] means highly strong correlation, |r|∈[.6,.8) means strong correlation, |r|∈[.4,.6) means moderate correlation, |r|∈[.2,.4) means weak correlation, |r|∈[0,.2) means very weak correlation or no correlation

*TT* total trials, *CT* correct trials, *TE* total number of errors, *PE* perseverative errors, *RE* random errors, *CC* the number of completed categories, *PE of CPT 3* perseverative errors of CPT 3, *RT of SCWT* reaction time of SCWT

*P < .05, **P < .01, the significance of the correlation coefficient

## Discussion

The most important finding in the present study was significant correlations between the negative factor scores and most neuropsychological assessments in patients with first-episode schizophrenia, with stronger correlations in adolescent patients. Most previous findings [22, 23] indicated that more attention should be paid to patients with first-episode schizophrenia who are drug naïve, to reduce the interference of drugs with symptoms and neurocognitive functions, and also to the differences between adolescence and adults.

On one hand, patients diagnosed with schizophrenia showed neurocognitive deficits in most assessments compared with healthy people, including executive function; visual search and processing speed; sustained, selective, and visual attention; and mental and cognitive flexibility. The present results were similar to many previous reports in the literature, which suggested that patients with schizophrenia showed deficits in executive function [31], visual attention, visual search speed, processing speed, mental flexibility [35] and selective attention [36]. That is to say, schizophrenia is a mental disorder that affects one’s neurocognitive function [2]. Some brain imaging examinations such as magnetic resonance tomography and positron emission tomography–computed tomography supported that abnormal functional areas in the brain of patients with schizophrenia may usually appear in the frontal lobes and temporal lobes [4]. The frontal lobe plays an important role in executive function, including the ability to forecast future outcomes resulting from present decisions, to determine better or worse choices, to deal with social emergencies, and to distinguish similarities and differences. The functions of temporal lobes cover reserving visual memories, processing sensory input, understanding language, keeping new memories and emotions, and extending in meaning. Some correlations may exist between abnormal activity of massive brain regions and neurocognitive function deficits in patients with schizophrenia [2]. Thus far, imaging examinations have been completed in some of these 149 subjects, and the details will be discussed in future studies. On the other hand, No significant difference was observed in clinical or neuropsychological assessments between adolescent and adult patients with first-episode schizophrenia, but some previous studies showed that adolescent patients with schizophrenia performed worse than adult patients in tasks of working memory, language, and motor function [21].

Significant correlations were obtained between negative symptoms and most neuropsychological assessments (CT, TE, PE, RE, and CC of WCST; CPT 1; TMT 1 and TMT 2; SCWT 1, SCWT 2, SCWT 3, and RT of SCWT) in patients with first-episode schizophrenia. It meant that the severity of negative symptoms was correlated with the deficits of executive function, sustained, selective, and visual attention, visual search and processing speed, and mental and cognitive flexibility in patients with schizophrenia [32, 33, 35, 36]. A previous similar study showed that negative symptoms were associated with neurocognitive function deficits involving executive function, sustained attention function [22], and cognitive function [23].

Most importantly, the present findings suggested closer correlations between the negative factor scores and some neuropsychological assessments outcomes in adolescent patients with first-episode schizophrenia, including more time spent in TMT 1 and TMT 2, fewer times of CPT 2, and a higher mean score of PE of WCST, compared with adult patients. Therefore, the severity of negative symptoms in adolescent patients with schizophrenia was more related to their neurocognitive deficits of visual, selective, and sustained attention; visual search and processing speed; mental flexibility; and executive function [36–38]. Some of the latest studies regarded schizophrenia as a collection of “neurodevelopmental disorders,” because it nearly emerged in late adolescence or early adulthood when the prefrontal cortex was still developing [2, 28–30]. The present results partly support the conclusion. Since adolescence is a key period of neurological and psychological development, earlier schizophrenia symptoms and neurocognitive deficits may both originate from earlier neurodevelopmental problems in adolescent patients with first-episode schizophrenia.

Hence, the negative symptoms of adolescent patients with schizophrenia are more closely related to their neurocognitive deficits, compared with adult patients with first-episode schizophrenia. For physicians, the negative symptom scores of PANSS become more important in evaluating neurocognitive deficits of adolescent patients with first-episode schizophrenia. Because it is easy for psychiatrists to obtain symptom characteristics from psychiatric examinations, PANSS scores, and description of patients or their family members, the physicians can view the severity of negative symptoms as a factor that infers neurocognitive deficits in patients with first-episode schizophrenia, especially adolescents. The evaluation of neurocognitive functions is complicated and time-consuming. First-visit adolescent outpatients with obvious negative symptoms may suggest serious neurocognitive deficits. Furthermore, the severity of negative symptoms can be widely used in evaluating the therapeutic effect.

The present study had several limitations. (1) The sample size was small. (2) The ratio of male and female participants was different between groups. (3) The study did not contain any follow-up data. (4) The study lacked neuroimaging examinations. (5) The cutoff age remained unclear. In this study, a total of 92 patients with schizophrenia were divided into two groups—33 adolescent patients with first-episode schizophrenia (whose age and age of onset were less than 18 years) and 59 adult patients with first-episode schizophrenia (whose age and age of onset were equal to or more than 18 years), which meant the cutoff age was 18 years. However, recent research has regarded schizophrenia to comprise four stages (i.e., stage 1: <12 years, risk stage; stage 2: 12–18 years, prodromal stage; stage 3: 18–24 years, psychosis stage; stage 4: >24 years, chronic disabled stage) [2]. Thus, it would be better that more cutoff points are set in future studies.

In summary, clinical negative symptoms are good indexes for evaluating neurocognitive deficits in patients with schizophrenia. In adolescent patients, doctors should pay more attention to negative symptom results of PANSS. Also, more brain imaging data should be collected to support the conclusions. Moreover, longer-term studies with a larger sample size are needed to draw more definite conclusions.

## Conclusions

1. Patients with first-episode schizophrenia show neurocognitive deficits in most neuropsychological assessments compared with healthy people.
2. The severity of negative symptoms in patients with first-episode schizophrenia is related to some of their neurocognitive deficits.
3. Most importantly, this relationship is even closer for adolescent patients with first-episode schizophrenia.

## Abbreviations

CC: Completed categories
CPT: Continuous performance test
CT: Correct trials
PANSS: Positive and negative syndrome scale
PE: Perseverative errors
RE: Random errors
RT: Reaction time
SCWT: Stroop color-word test
TE: Total number of errors
TMT: Trail making test
TT: Total trials
WCST: Wisconsin card sorting test
